# Clinical characteristics and outcomes of patients admitted with acute heart failure: insights from a single-center heart failure registry in South India

**DOI:** 10.1186/s43044-021-00161-w

**Published:** 2021-05-01

**Authors:** Aashiq Ahamed Shukkoor, Nimmy Elizabeth George, Shanmugasundaram Radhakrishnan, Sivakumar Velusamy, Rajendiran Gopalan, Tamilarasu Kaliappan, Premkrishna Anandan, Ramasamy Palanimuthu, Vidhyakar Balasubramaniam, Vinoth Doraiswamy, Arun Kaushik Ponnusamy

**Affiliations:** 1 Department of Cardiology, PSG Institute of Medical Sciences and Research, Coimbatore, Tamilnadu India; 2 Department of Pharmacy Practice, PSG College of Pharmacy, Coimbatore, Tamilnadu India

**Keywords:** Heart failure, Registry, Mortalities, In-hospital

## Abstract

**Background:**

The epidemiology of HF in India is largely unexplored. Current resources are based on a few hospital-based and a community-based registry from North India. Thus, we present the data from a single hospital-based registry in South India. Patients admitted with acute heart failure over a period of 1 year were enrolled in the registry and were characterized based on their ejection fraction (EF) measured by echocardiogram. The clinical profile of the patients was assessed, including their in-hospital outcomes. One-way ANOVA and univariate analysis were performed for comparison between three EF-based groups and for the assessment of in-hospital outcomes.

**Results:**

A total of 449 patients were enrolled in the registry, of which 296, 90, and 63 patients were categorized as, HFrEF, HFmrEF, and HFpEF, respectively. The prevalence of HFrEF was higher (65.99%). The mean age (SD) of the study cohort was 59.9±13.3. The majority of the patients presented with acute denovo HF (67%) and were more likely to be males (65.9%). The majority of patients presented with warm and wet clinical phenotype (86.4%). In hospital mortality was higher in HFmrEF (3.3%).

**Conclusion:**

Patients with HFrEF had high adherence to guideline-directed medical therapy (GDMT). HFrEF patients were also likely to have longer hospital stay along with a worsening of renal function. The in-hospital mortality was comparable between the EF-based groups. Additionally, the association of clinical phenotypes with outcome highlighted that patients in warm and wet phenotype had a longer length of hospital stay, whereas the mortality and worsening renal function rates were found to be significantly higher in the cold and wet group.

## Background

Heart failure is a clinical syndrome which is progressive in nature. A rise in cardiovascular risk factors has led to a perpetual escalation in the prevalence and incidence of HF [1]. Additionally, the presence of myriads of comorbid conditions associated with HF imposes a major economic burden [2]. Despite significant developments in the management of chronic HF, it still remains a public health issue with worse prognosis leading to several million hospitalizations worldwide [3, 4]. According to the INDUS study, the estimated prevalence of HF in India in 2016 was 1% of the total population; that is about 8 to 10 million patients. The HF burden in India based on the existing evidence is alarming [5].

At present, the understanding of HF burden and management is based on western guidelines, with poor representation of low-resource countries. Contemporary HF data from developing and low-middle income countries are sparse, and the epidemiology of HF in India is largely unexplored. Current resources are based on a few hospital-based registries and a community-based registry [5–9]. We present the data from a single hospital-based registry in South India in order to compare the causes of hospitalization, clinical presentation, management strategies, and outcome with other national and international registries. The data could be used for identification of key factors of HF hospitalization in India and development of native guidelines in low-resource countries.

The prime objectives of this registry were (i) to describe the precipitating factors for hospitalization and the clinical characteristics of HF patients, (ii) to describe the diagnosis and pharmacological strategies of management in HF patients, and (iii) to describe the in-hospital outcome of patients admitted with HF.

## Methods

We initiated a prospective, single-center hospital-based registry of patients admitted with heart failure from July 2018 to July 2019 at PSG hospitals, Coimbatore. The study was approved by the institutional human ethics committee (19/275), Coimbatore, India. This registry was established as a part of the quality improvement program and to monitor the in-hospital outcomes of HF patients.

Data was collected systematically by clinical pharmacist, a member of the multidisciplinary HF team. A total of 449 patients were enrolled. Precipitating factors for decompensation were documentedby means of a structured interview of patients or their caretakers. Patients were diagnosed with HF based on signs, symptoms, echocardiography, and natriuretic peptide levels if available. Eligible patients were categorized based on left ventricular ejection fraction (LVEF) into heart failure with reduced ejection fraction (LVEF <40%), mid–range ejection fraction (LVEF 41–49%), and preserved ejection fraction (≥50%), measured using the Simpson method. The study cohort was also classified based on clinical phenotype, according to Forrester’s classification to determine the in-hospital outcome in each of the phenotype.

The clinical outcomes of patients during their course in hospital such as the length of stay, in-hospital mortality, NYHA class at discharge and worsening renal function were assessed. Worsening renal function was defined as a rise in serum creatinine levels by more than 30% from baseline. The aforementioned clinical outcome parameters were analyzed across the entire spectrum of LVEF. Clinical outcome parameters were categorized based on clinical phenotype of patients as well.

### Inclusion and exclusion criteria

All patients admitted with HF irrespective of etiology and age more than 18 years were enrolled in the registry.

### Data collection

Patient’s demographic details, medical history, co-morbidities, risk factors, and presenting signs and symptoms were included in the data collection. Diagnostic information like laboratory parameters and details of invasive and non-invasive diagnostic procedures were recorded. The prevalence of anemia across the subcategories was analyzed in this study wherein anemia was defined as hemoglobin <12g/dl in females and <13g/dl in males, as defined by the World Health Organization. The serum iron level was evaluated for all patients with anemia and also for all affordable patients without anemia. Iron deficiency was defined as ferritin <100 μg/l or ferritin of 100–299 μg/l with a transferrin saturation<20%.

Echocardiographic details like left ventricular systolic and diastolic function, details of valvular abnormalities, the presence of pulmonary hypertension, and right ventricular function were registered.

Pharmacological management of patients, such as inclusion of neurohormonal blockers and other supportive medications was included. The precipitating factors for acute decompensation were captured. Invasive procedures during the hospital admission like percutaneous coronary intervention (PCI), coronary artery bypass grafting (CABG), intra-aortic balloon pump (IABP), implantable cardio-defibrillator (ICD), cardiac resynchronization therapy (CRT), and pacemaker implantation (PPI) were recorded.

### Statistical analysis

Continuous variables were presented as median and categorical variables were presented as percentages. Univariate analysis was performed to identify the associationof three EF groups within hospital mortality. In univariate analysis, the hazard ratio (HR) with 95% confidence intervals (CI) was calculated, respectively. Statistical significance was defined as *p* values < 0.05. SPSS 24.0 (IBM Corporation, New York, USA) was used for all statistical analyses.

## Results

A total of 449 patients admitted with HF under the Department of Cardiology during the study period (July 2018 to July 2019) were enrolled in the registry. Patients were stratified based on their EF determined by echocardiography. HFrEF constituted 65.9% of patients, while HFmrEF and HFpEF made up to 20% and 14.03% of the patients admitted with heart failure, respectively. About 67% of patients presented with acute de novo heart failure, while 32.9% of patients presented with acute decompensated heart failure.

The mean age (SD) of the study population was 59.9±13.3 years. The mean age of patients in all the three EF-based groups were similar with no statistically significant difference. Among the total enrollees, 296 (65.9%) were males and 153 (34.9%) were females. Both HFrEF and HFmrEF groups showed a male predominance (71.62% vs.61.1%) whereas HFpEF group had a marginally higher number of female patients (53.9%) (*p*<0.05).

Type 2 diabetes mellitus was the most prevalent comorbidity in HFrEF and HFmrEF groups (62.8% and 56.6%) (*p*<0.001), whereas systemic hypertension was the most prevalent comorbidity in the HFpEF group. Coronary artery disease (CAD), chronic obstructive pulmonary disease (COPD), and chronic kidney disease (CKD) were the other relevant comorbiditiesin the study population, as elaborated in Table 1.

**Table 1:** Baseline characteristics of patients admitted with heart failure

Variables	Total *n*=449	HFrEF *n*=296 (65.9%)	HFmrEF *n*=90 (20%)	HFpEF *n*=63 (14.03%)	*p* value
Age	59.9±13.3	59.1±13.6	61.2±11.7	61.3±13.5	0.212
Sex					
Male	296 (65.9)	212 (71.62)	55 (61.1)	29 (46.0)	**0.000**
Female	153 (34.0)	84 (28.3)	35 (38.8)	34 (53.9)	**0.000**
Co morbidities					
Diabetes	259 (57.6)	186 (62.8)	51 (56.6)	22 (34.9)	**0.000**
Hypertension	233 (51.8)	158 (53.3)	45 (50)	30 (47.6)	0.694
Obesity	32 (28.5)	24 (8.1)	4 (4.4)	4 (6.3)	0.482
CKD	60 (13.3)	44 (14.8)	12 (13.3)	4 (6.3)	0.212
COPD	35 (7.7)	26 (8.7)	5 (5.5)	4 (6.3)	0.547
CAD	236 (52.5)	180 (60.8)	46 (51.1)	10 (15.8)	**0.000**
Hepatic dysfunction	50 (11.1)	43 (14.5)	6 (6.66)	1 (1.5)	**0.004**
Past medical history					
PCI	37 (8.2)	30 (10.1)	6 (6.6)	1 (1.5)	0.068
CABG	26 (5.7)	21 (7.0)	4 (4.4)	1 (1.5)	0.197
Valve procedures	19 (4.2)	4 (1.3)	4 (4.4)	11 (17.4)	**0.000**
CRT/ICD	9 (2)	7 (2.3)	1 (1.1)	1 (1.5)	0.736
H/o hospitalization for HF	79 (17.5)	59 (19.9)	11 (12.2)	9 (14.2)	0.264
Presentation					
Acute denovo HF	301 (67)	170 (57.4)	72 (80)	59 (93.6)	**0.000**
Acute decompensated HF	148 (32.9)	126 (42.5)	18 (20)	4 (6.3)	**0.000**
Presentation					
Acute denovo HF	301 (67)	170 (57.4)	72 (80)	59 (93.6)	**0.000**
Acute decompensated HF	148 (32.9)	126 (42.5)	18 (20)	4 (6.3)	**0.000**
Presenting symptoms					
Pulmonary congestion	416 (92.6)	283 (95.6)	81 (90)	52 (82.5)	**0.000**
Angina	48 (10.6)	34 (11.4)	8 (8.8)	6 (9.5)	0.745
Syncope/presyncope	4 (0.8)	2 (0.6)	0	2 (3.1)	0.096
Palpitation	73 (16.2)	48 (16.2)	13 (14.4)	12 (19.0)	0.751
Pedal edema	362 (80.6)	253 (85.4)	68 (75.5)	41 (65.0)	**0.001**
Weight gain	290 (64.5)	241 (81.4)	30 (33.3)	19 (3.0)	**0.005**
Vitals					
Heart rate^#^ (bpm)	94.7±22.8	95.9±22.1	96±23.8	88.2±24.3	**0.041**
SBP^#^ (mmHg)	121.17±25.1	119.3±25.2	126±24.3	124.8±25.4	0.079
Increased JVP	334 (74.3)	241 (81.4)	57 (63.3)	36 (57.1)	**0.000**
Electrocardiogram (ECG)					
Left bundle branch block	43 (9.5)	31 (10.4)	8 (8.8)	4 (6.3)	0.579
Sinus rhythm	362 (80.6)	255 (86.1)	65 (72.2)	42 (66.6)	**0.000**
ECHO					
Ejection Fraction	37.8 ±10.6	32.02±5.9	43.3±2.9	57.7±5.3	**0.000**
Grade I diastolic dysfunction	44 (9.7)	19 (6.4)	12 (13.3)	13 (20.6)	**0.000**
Grade II diastolic dysfunction	71 (1.5)	49 (16.5)	11 (12.2)	11 (17.4)	**0.000**
Grade III diastolic dysfunction	149 (33.1)	116 (39.1)	23 (25.5)	10 (15.8)	**0.000**
Left ventricular hypertrophy	11 (2.4)	8 (2.7)	1 (1.1)	2 (3.1)	0.642
Right ventricular dysfunction	15 (3.3)	6 (2.0)	3 (3.3)	6 (9.5)	0.011
Mitral regurgitation	289 (64.3)	209 (70.6)	50 (55.5)	30 (47.6)	**0.000**
Tricuspid regurgitation	115 (25.6)	85 (28.7)	23 (25.5)	7 (11.1)	**0.013**
RVSP	50.8 ±15.1	50.1±12.8	51.2±15.3	53.7±23.1	0.464
Pericardial effusion	93 (20.7)	67 (22.6)	20 (22.2)	6 (9.5)	**0.000**
Laboratory values					
Haemoglobin^#^ (g/dL)	12.03 ±2.2	12.2±2.0	11.5±2.5	11.5±2.4	**0.015**
- Male (<13g/dl)	144 (32)	100 (33.7)	27 (30)	17 (27)	0.518
- Female (<12g/dl)	43 (9.5)	4 (1.3)	23 (25.5)	16 (25.3)	0.000
eGFR (ml/min/1.73m^2^)					
- *Admission*	61.9±32	59.8±30.8	60.5±30.9	73.6±36.4	**0.008**
- *Peak*	55.5±29.4	53.5±28.2	53.5±28.8	67.2±33.3	**0.003**
- *Discharge*	62.±34.8	60.6±35.2	60±32.6	71.2±34.9	0.080
Renal dysfunction					
- Male (Cr >1.4mg/dl)	89 (19.8)	68 (22.9)	15 (16.6)	6 (9.5)	**0.036**
- Female (Cr >1.2mg/dl)	46 (10.2)	28 (9.4)	12 (13.3)	6 (9.5)	0.559
NT-proBNP	8020±9835	7467.7±8381[45]	13662 ±1431 0[10]	3788±4810. 6[11]	**0.004**
Hyponatremia	10 (2.2)	8 (2.7)	2 (2.22)	0	0.420
Hypokalemia	92 (20.4)	70 (23.6)	10 (11.1)	12 (19.0)	**0.034**
Hyperkalemia	34 (7.5)	21 (7.0)	9 (10)	4 (6.3)	0.416
Etiology					
IHD	215(47.8)	173 (58.4)	29 (32.2)	13 (20.6)	0.951
Non ischemic	171(38)	135 (45.6)	15 (16.6)	21 (33.3)	**0.000**
Clinical phenotype					
Warm & dry	32 (7.1)	9 (3.0)	3 (3.3)	20 (31.7)	**0.000**
Warm & wet	388 (86.4)	259 (87.5)	86 (95.5)	43 (6.8)	**0.000**
Cold & dry	1 (0.2)	1 (0.3)	0	0	0.773
Cold & wet	28 (7.4)	27 (9.1)	1 (1.1)	0	**0.002**
Interventions					
PCI	9 (2.0)	8 (2.7)	0	1 (1.5)	0.270
CABG	8 (1.7)	5 (1.6)	2 (2.22)	1 (1.5)	0.938
IABP	2 (0.4)	2 (0.6)	0	0	0.597
ICD	1 (0.2)	1 (0.3)	0	0	0.773
CRT	2 (0.4)	2 (0.6)	0	0	0.597
PPI	5 (1.1)	2 (0.6)	2 (2.22)	1 (1.5)	**0.000**

*CKD* chronic kidney disease, *COPD* chronic obstructive pulmonary disease, *CAD* coronary artery disease, *PCI* percutaneous coronary intervention, *CABG* coronary artery bypass graft, *CRT* cardiaresynchronization therapy, *ICD*implantable cardioverter defibrillator, *HF* heart failure, *SBP* systolic blood pressure, *DBP* diastolic blood pressure, *JVP* jugular venous pressure, *RVSP* right ventricular systolic pressure, *NTproBNP N*-terminal pro-brain natriuretic peptide, *IHD* ischemic heart disease, *PCI* percutaneous coronary intervention, *CABG* coronary artery bypass graft, *IABP* intra-aortic balloon pump, *ICD* implantable cardioverter defibrillator, *CRT* cardiac resynchronization therapy, *PPI* permanent pace-maker implantation, *eGFR* estimated glomerular filtration rate

Mean admission systolic blood pressure was 119.3±25 mmHg in patients with HFrEF, 126±24.3 mmHg, and 124.8±25.4 mmHg in patients with HFmrEF and HFpEF, respectively. While breathlessness was the most common presenting symptom in HFrEF and HFpEF group, tiredness and weight gain were the most presenting symptoms in HFmrEF group.

The prevalence of left bundle branch block was the highest in the HFrEF group (10.4%). Patients with HFpEF were found to have a higher incidence of right ventricular dysfunction (9.5%) in comparison to HFmrEF and HFpEF groups. Mitral regurgitation and tricuspid regurgitation were more often detected in patients with HFrEF (70.6%, 28.7%). The mean left ventricular ejection fraction of patients with HFrEF was 32.02±5.9, 43.3±2.9 in HFmrEF and 57.7±5.3 in HFpEF. The mean right ventricular systolic pressure (RVSP) of the study population was 50.8±15.1. There was no statistical difference in the RVSP of patients in the three EF-based subgroups (*p*>0.05).

The prevalence of anemia in our cohort was 41.6%. More than half of the patients in HFmrEF and HFpEF groups were found to have anemia (55.5% and 52.3%), while anemia prevailed in 35.13% of patients in the HFrEF group. Among HF patients with anemia, iron deficiency was prevalent in 93.6%. Of patients without definite anemia, serum iron levels could be estimated only in 24.9% due to reasons of unaffordability wherein 30.63% of patients were found to have low serum iron level. Iron was substituted in intravenous form as ferric carboxymaltose in all patients with iron deficiency.

A significantly higher proportion of patients with HFrEF had presented with a baseline renal dysfunction due to CKD or AKI, when compared to other subgroups. Natriuretic peptide (NT-proBNP) levels were available only in 17.8% of the total patients. In patients with HFrEF, the mean±SD values were 7467.7±8381. In HFmrEF and HFpEF groups, the mean±SD values were 13662±14310 and 3788±4810.6, respectively.

Hypokalemia was the most common electrolyte abnormality across all the EF-based subgroups; the majority being in HFrEF group (23.6%) followed by 11.1% and 19% in HFmrEF and HFpEF groups, respectively. About 34 patients were noted to have hyperkalemia in the study cohort, among which, HFmrEF group had higher number of patients with raised potassium levels (10%). Sodium levels were available in 132 patients, of which 10 patients (7.57%) were observed to have hyponatremia.

The study cohort was classified based on clinical phenotype, according to Forrester’s classification. A large share of the patients in the study population presented with congestion without hypoperfusion (warm and wet phenotype) (86.4%). This finding was consistent with HFrEF group wherein majority of patients were admitted with warm and wet (87.5%) followed by 9.1% of patients with cold and wet and 3% of patients in warm and dry phenotype. The HFmrEF group also had the highest number of patients in warm and wet category (95.5%), followed by 3.3% of patients in warm and dry type. The HFpEF group was unique in terms of clinical phenotype; with the majority of patients being presented with warm and dry phenotype (31.7%) followed by the warm and wet phenotype (6.8%).

**Fig. 1:**
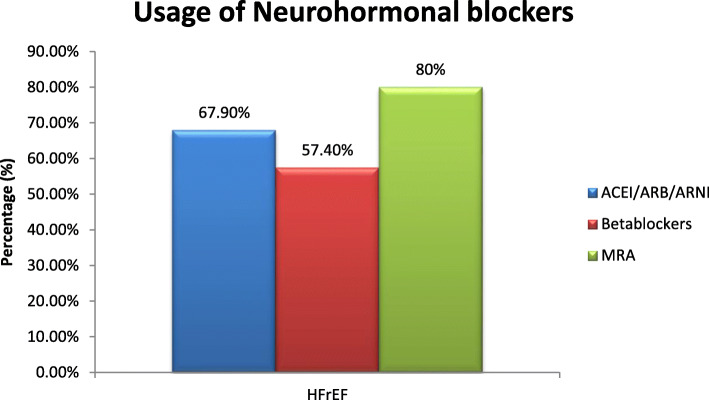
Usage of neurohormonal blockers. ACEI angiotensin-converting enzyme inhibitor, ARB angiotensin receptor blocker, ARNI angiotensin receptor neprilisin inhibitor, MRA minerelocorticoid reeceptor antagonist

The usage of guideline directed medical therapy (GDMT) was analyzed in the HFrEF group. Angiotensin-converting enzyme inhibitors (ACEI)/angiotensin receptor blockers (ARB)/angiotensin receptor neprilysin inhibitors (ARNI) was prescribed in 67.9% of patients, beta blockers in 57.4% of patients, and mineralocorticoid receptor antagonist (MRA) was used in 80% of the HFrEF patients enrolled in the registry (Fig. 1).

Three out of four patients received intravenous diuretics and 1 in 10 patients received inotropes. It was observed that noradrenaline was the most commonly used inotrope in the study population (21 patients) of which, 18 patients were admitted with HFrEF, and 3 patients were admitted with HFmrEF. Intravenous diuretics were used in 86.4% of patients in HFrEF group, 64.4% in HFmrEF, and 52.3% in HFpEF group. All the patients in the study population were prescribed oral diuretics at discharge.

The common interventions underwent by the study population were PCI (2%), followed by CABG (1.7%). ICD implantation was done in 2 patients, and CRT was done in 1 patient in the HFrEF group.

Dietetic non-compliance was found to be the major precipitating factor for hospital admission in the study cohort across all the groups (69.4%). This was followed by drug non-compliance (31.6%). The other contributing factors were found to be acute coronary syndrome (24.4%) anemia (17.3%) and arrhythmias (16.7%) (Table 2).

**Table 2:** Precipitating factors for heart failure

	Total *n*=449	HFrEF *n*=296 (65.9%)	HFmrEF *n*=90 (20%)	HFpEF *n*=63 (14.03%)	*p* value
Drug non-compliance	190 (42.3)	142 (31.6)	28(31.1)	20(31.7)	**0.001**
Increased fluid/salt intake	418 (93)	312 (69.4)	80(88.8)	26(36.5)	0.089
Acute Infections	55 (12.2)	41 (9.1)	12(13.3)	2(3.1)	0.092
Arrhythmias	75 (16.7)	56 (12.4)	15(16.6)	4(6.3)	**0.000**
ACS	110 (24.4)	82 (18.2)	16(17.7)	12(19.0)	**0.000**
Acute valvular pathology	55 (12.3)	41 (9.1)	8(8.8)	6(9.5)	**0.000**
Thyrotoxicosis	16 (3.5)	12 (2.6)	2(2.2)	2(3.1)	0.122
Accelerated hypertension	19 (4.2)	14 (3.1)	2(2.2)	3(4.7)	**0.017**
Pulmonary thromboembolism	3 (0.6)	2 (0.4)	1(1.1)	0	0.773
Anemia	78 (17.3)	58 (12.9)	15(16.6)	5(7.9)	**0.043**

*ACS* acute coronary syndrome

The mean (SD) length of hospital stay of the study population was 8.12 days (3.28) about 68.5% of patients in HFrEF group had a hospital stay of more than 5 days, while 26.6 % and 33.3% of patients in HFmrEF and HFpEF group had above 5 days of hospital stay.

The NYHA functional class of heart failure patients assessed at the time of discharge. It was found that 6.6% with HFmrEF group had NYHA class IV symptoms at discharge, followed by 4% of patients with HFrEF. Worsening renal function in hospital was noted the most in HFrEF group (39.8%) followed by 36.6% in HFmrEF and 26.9% in HFpEF groups (*p*<0.05). The in-hospital mortality in HFmrEF (3.3%) was twice that of HFrEF and HFpEF groups, but this difference was not statistically significant (*p*>0.05) (Table 3).

**Table 3:** In-hospital outcome: based on ejection fraction (univariate analysis)

	Total *n*=449 (%)	HFrEF *n*=296(65.9%)	HFmrEF *n*=90(20%)	HFpEF *n*=63(14.03%)	*p* value
In-hospital mortality (cardiac)	9 (2)	5 (1.6)	3 (3.3)	1 (1.5)	0.137
Length of in-hospital stay (days) [>5days]	248(55.2)	203(68.5)	24(26.6)	21(33.3)	**0.000**
NYHA IV at discharge	18 (4)	12 (4)	6 (6.6)	0	0.890
WRF at discharge [>30% from baseline]	168 (37.4)	118 (39.8)	33 (36.6)	17 (26.9)	0.042

*NYHA* New York Heart Association, *WRF* worsening renal function

The in-hospital outcome of the patients were analysed with respect to their clinical phenotype. It was noted that patients in cold and wet group (25%) recorded the highest mortality rates followed by warm and wet group (0.5%). The length of hospital stay of more than 5 days was highest in warm and wet category (60.3%). NYHA class IV at the time of discharge were maximum in the cold and wet group (14.2%). Worsening of renal function during the course in hospital was highest in cold and wet phenotype (53.5%) (Table 4).

**Table 4:** In-hospital outcome: based on clinical phenotypes (univariate analysis)

	Total *n*=449 (%)	Warm & dry *n*=32 (7.1%)	Warm & wet *n*=388 (86.4%)	Cold & dry *n*=1 (0.2%)	Cold & wet *n*=28 (6.2%)	*p* value
In-hospital mortality (cardiac)	9 (2)	0	2 (0.5)	0	7 (25)	**0.000**
Length of in-hospital stay (days) [>5days]	248 (55.2)	4 (12.5)	234 (60.3)	0	10 (35.7)	**0.000**
NYHA IV at discharge	18 (4)	0	14 (3.6)	0	4 (14.2)	0.087
WRF at discharge [>30% from baseline]	170 (37.8)	8 (25)	147 (37.8)	0	15 (53.5)	**0.001**

*NYHA* New York Heart Association, *WRF* worsening renal function

**Fig. 2:**
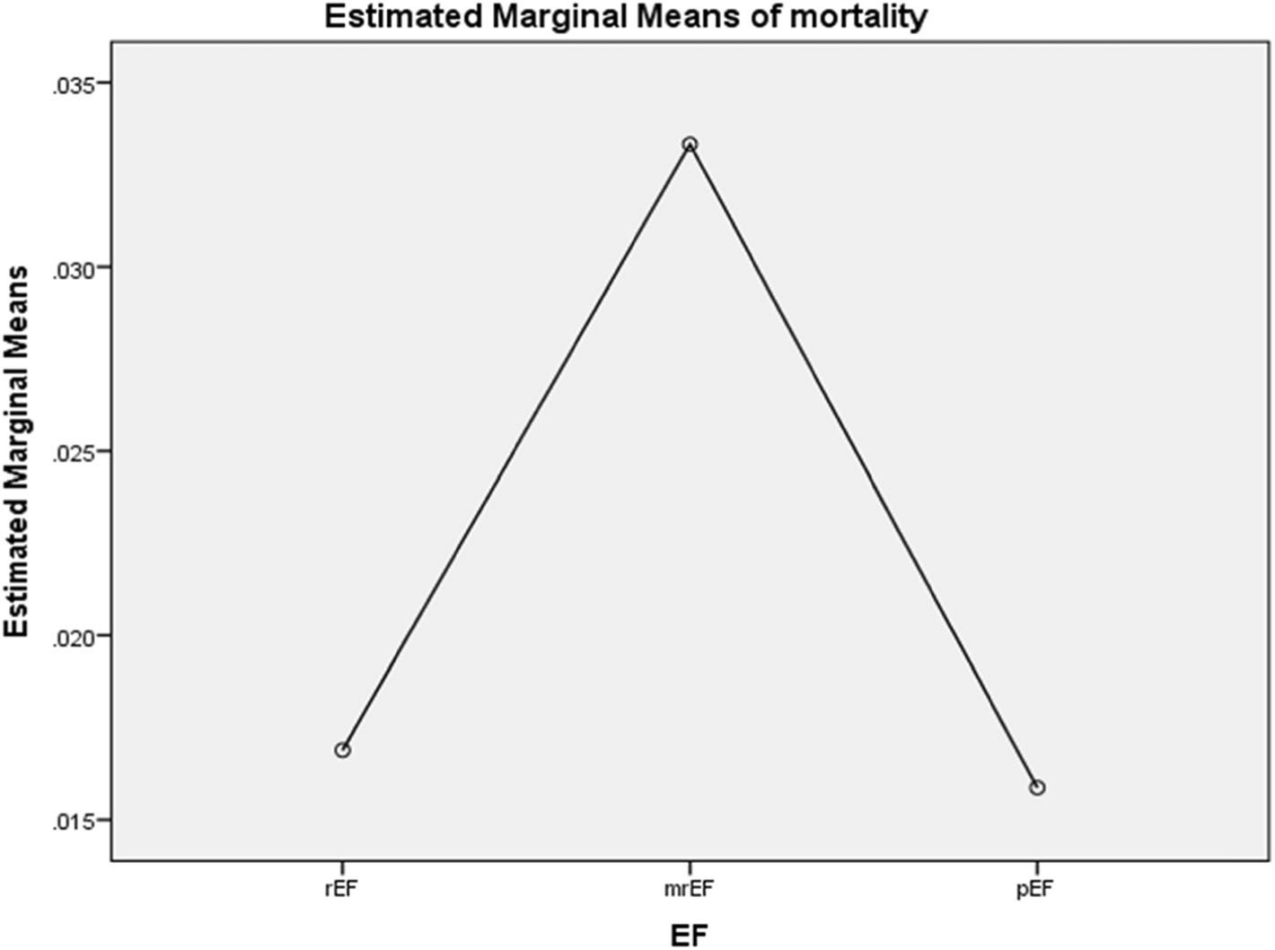
Univariate analysis for mortality in patients admitted with heart failure. rEF reduced ejection fraction, mrEF mid-range ejection fraction, pEF preserved ejection fraction

Univariate Cox analysis showed that patients with HFmrEF when compared to HFpEF and HFrEF was associated with increased mortality, but statistically not significant [*p*>0.005] (Fig. 2). Length of in-hospital stay was increased in HFrEF, compared to HFmrEF and HFpEF [*p* = 0.000]. In comparison with clinical phenotypes, cold and wet category had increased in-hospital mortality and worsening renal function compared to other categories [*p*=0.000 and *p*=0.001, respectively], but the length of in-hospital stay was increased in with warm and wet phenotype [*p*=0.000].

## Discussion

Our registry is a prospective observational analysis of heart failure patients from a single center in south India over a period of 1 year. Most available HF registries have classified patients based on the etiology or with respect to clinical presentation. Formerly, HF patients were classified based on LVEF as HFrEF and HFpEF. The 2016 ESC guidelines included HFmrEF as a distinct group to promote research in this gray area due to paucity of data [12]. The novelty of this registry lies in the projection of demographics, clinical characteristics, pharmacological management, and in-hospital outcome characteristics in LVEF-based subgroups as well as clinical phenotype-based subgroups.

As single-centered registries are limited and inconsistent, we compared our findings with multicenter registries. In comparison to other international cohorts, our study population was relatively a decade younger (59.9±13.3) [10, 13–16] and was comparable to THFR, a south Indian registry (61.2±13.7) and Indian subcontinent AHF patients in the Gulf acute heart failure registry (54±11) [4, 6]. The younger cohort in the Indian subset could possibly be due to higher prevalence of ischemic heart disease at a younger age in this geographical region, which might predispose for the development of HF [17].

HFrEF and HFmrEF made a substantial portion of the study population. The higher prevalence of HFrEF and HFmEF could be explained by the setting of our study, being hospital inpatients as opposed to HF registries [18] which included patients from outpatient clinics.

The study population predominantly comprised of male patients. This finding was in contrast to the USA-based ADHERE registry whereas it was in line with THFR and AFAR registries conducted in India [6, 7, 15]. This outlines increased risk factors for ischemic heart disease in males, leading to HF [19]. Male preponderance were higher in HFrEF and HFmrEF whereas the female dominance was higher in HFpEF group, which is in accordance with previous data [20].

A striking difference in the clinical presentation of patients was observed. Most of the patients in this registry presented with acute de novo HF. This finding was similar to the Indian cohort in an ethnic-based comparative study in HF patients conducted in the Middle East [4]. Our finding contradicts THFR in which only 39.8% of HF hospitalization was recorded to be acute de novo heart failure [6].

The burden of diabetes mellitus is high in South India and is a major risk factor for developing HF due to diffuse multivessel disease, recurrent MI, etc. [11]. Type II diabetes mellitus was thus unsurprisingly the most common comorbidity in patients with HFrEF and HFmrEF. As foreseen, hypertension was the most common comorbidity in HFpEF patients, which was in consensus to the study conducted by LyuSiqi, which evaluated the clinical characteristics and prognosis of HF based on LVEF [21].

CAD was the most common comorbidity across the entire spectrum of HF patients. CAD alone was noted in 52.5% of the patients, which was comparable to several international multicenter registries in which overall prevalence ranged from 40–61% [14, 15, 20]. It highlights the global IHD burden which could lead to cardiac remodeling and further progression to heart failure [22].

Echocardiographic findings in our registry showed a higher incidence of LVH and RV dysfunction in HFpEF group compared to other patients. These findings confirmed the fact that left ventricular hypertrophy due to systemic hypertension is an important cause for diastolic dysfunction and HFpEF [23].

The prevalence of anemia in the study cohort was 41.6%, which was more than comparable cohorts. The Swedish HF registry [24] reported a prevalence of anemia in 34% of the population and the HF registry of the “Get with the guideline” population [25] reported anemia in only 14% of the population. In this registry, anemia was more common in HFmrEF group. This contradicts the findings of previous research which estimated higher prevalence of anemia in higher EF [24, 25]. Importantly, iron deficiency was the most common cause of anemia in the study cohort (93.6%). Among the 24.9% of patients without anemia, iron deficiency was prevalent in 30.63%, which was comparable to another hospital-based observational study of the anemia profile in HF patients in India [26].

Although, not significant, hyponatremia and hypokalemia were observed in the study population were higher in the HFrEF group which could be attributed to the aggressive intravenous diuretic use (*p*>0.05), whereas hyperkalemia was found to be higher in the HFpEF group (*p*=0.416).

Dietetic and therapeutic non-compliance was the most common precipitating factor for HF hospitalization in the entire study population. This finding reflects the need of multidisciplinary chronic disease management clinics and patient counseling to reinforce medication and dietary adherence.

It was found that the usage of guideline-directed medical therapy (GDMT) in HFrEF patients in our study higher than comparable cohort in India [6, 27]. A multidisciplinary HF team and an on-going clinical audit of discharge prescriptions for evidence-based medication in HF have been proven to uplift the quality of HF management, which is being adopted at our center [28].

About 13.1% of patients in the HFrEF group and 7.7% of patients in the HFmrEF group were initiated on inotropes, whereas none of the patients in the HFpEF group had undergone inotropic treatment. The usage of inotrope in hospital was similar to THFR [6].

India, being a low-middle income country, ICD and CRT rates in the study population were minimal as opposed to other HF registries. Though eligible patients were offered device therapy, because of financial reasons the number of patients who underwent device therapy was noted to be low in our registry.

The overall mortality rate of HF patients over a period of 1 year in this study was 2%, opposed to higher mortality rates in several previously published data. The in-hospital mortality was comparable in all the three EF based subgroups (*p*>0.05). This result is inconsistent with previous research in which the prognosis of the HFmrEF group was intermediate to HFrEF and HFpEF [29, 30].

Reduced EF was associated with longer length of hospital stay (*p*=0.000); probably due to higher fluid overload requiring aggressive intravenous diuretic use, diuretic resistance, and time required to stabilize patients on GDMT. The difference between the LVEF-based subgroups in terms of NYHA IV symptoms at the time of discharge was statistically not significant (*p*>0.05). Worsening renal function at the time of discharge of the HF patients in our study was significantly more in HFrEF group (*p*=0.042). This reflects diuretic or ACEI-induced acute kidney injury and higher incidence of hypoperfusion in this subgroup.

Association of in-hospital mortality with the clinical phenotype of patients showed a higher rate of in-hospital deaths in cold and wet phenotype (*p*=0.000), confirming that hypoperfusion is a marker of HF severity is associated with poor outcomes [31, 32]. Length of stay in the hospital significantly exceeded in patients with warm and wet phenotype (*p*=0.000), which can be attributed to the intravenous diuretic use. Worsening renal function in the hospital was also noted to be higher in cold and wet phenotype (*p*=0.001) probably due to associated hemodynamic changes. The NYHA IV symptoms among patients did not vary significantly between the clinical phenotype-based subgroups.

This study has limitations inherent to most observational studies. It was a single-center study and data was collected by a single observer. We failed to comprehensively compare our findings with other indian hospital-based registries, and as to date, there is no single center or multicentre national registries in India that has elaborated on clinical characteristics and outcomes of patients based on LVEF and clinical phenotype. Details about a target dose of mandated drugs and follow-up data of patients were not extracted in this study. Cardiac rehabilitation of in-hospital patients, including 6 minute walk distance and cardiopulmonary exercise test (CPET) were not feasible in all patients; hence, the data were not included in the registry.

## Conclusion

This prospective HF registry exhibits systematic characterization of patients admitted with HF. We have elaborated the causes of decompensation, clinical characteristics, management, and in-hospital outcome of HF patients based on their LVEF and clinical phenotype. There is a striking disparity in the aforementioned parameters across the EF-based subgroups when compared to other international cohorts. Our study thus highlights the pressing need of native HF guidelines in low-resource countries in order to address population-specific problems in the prevention and management of HF.

## Data Availability

The manuscript data is available on request to the corresponding author.

## Abbreviations

HF: Heart failure
EF: Ejection fraction
HFrEF: Heart failure with reduced ejection fraction
HFmrEF: Heart failure with midrange ejection fraction
HFpEF: Heart failure with preserved ejection fraction
GDMT: Guideline disrected medical therapy
LVEF: Left ventricular ejection fraction
PCI: Percutaneous coronary intervention
CABG: Coronary artery bypass grafting
IABP: Intra-aortic balloon pump
ICD: Implantable cardioverter defibrillator
CRT: Cardiacresynchronization therapy
PPI: Permanent pacemaker implantation
CAD: Coronary artery disease
CKD: Chronic kidney disease
COPD: Chronic obstructive pulmonary disease
ACEI: Angiotensin-converting enzyme inhibitor
ARB: Angiotensin receptor blocker
ARNI: Angiotensin receptor neprilysin inhibitor
MRA: Minerelocorticoid receptor antagonist
NYHA: Newyork heart assoaciation
CPET: Cardiopulmonary exercise test

## Declarations

The submitted article is an original article and has not been published before.
